# Pretreatment with Warfarin Attenuates the Development of Ischemia/Reperfusion-Induced Acute Pancreatitis in Rats

**DOI:** 10.3390/molecules25112493

**Published:** 2020-05-27

**Authors:** Dawid Maduzia, Piotr Ceranowicz, Jakub Cieszkowski, Krystyna Gałązka, Beata Kuśnierz-Cabala, Zygmunt Warzecha

**Affiliations:** 1 Department of Physiology, Faculty of Medicine, Jagiellonian University Medical College, 31-531 Cracow, Poland; dawid.maduzia@uj.edu.pl (D.M.); jakub.cieszkowski@uj.edu.pl (J.C.); mpwarzec@cyf-kr.edu.pl (Z.W.); 2 Department of Anatomy, Faculty of Medicine, Jagiellonian University Medical College, 31-034 Cracow, Poland; 3 Department of Pathology, Faculty of Medicine, Jagiellonian University Medical College, 31-531 Cracow, Poland; krystyna.galazka@uj.edu.pl; 4 Department of Diagnostics, Chair of Clinical Biochemistry, Faculty of Medicine, Jagiellonian University Medical College, 31-501 Cracow, Poland; mbkusnie@cyf-kr.edu.pl

**Keywords:** acute pancreatitis, inflammation, coumarin, warfarin, fibrin, pancreatic blood flow, biochemical indicators

## Abstract

In acute pancreatitis (AP), pancreatic damage leads to local vascular injury, manifesting as endothelial damage and activation, increased vascular permeability, leukocyte rolling, sticking and transmigration to pancreatic tissue as well as activation of coagulation. Previous studies have shown that pretreatment with heparin or acenocoumarol inhibits the development of AP. The aim of the present study was to check the impact of pretreatment with warfarin, an oral vitamin K antagonist, on the development of ischemia/reperfusion-induced AP in rats. AP was induced by pancreatic ischemia followed by reperfusion of the gland. Warfarin (90, 180 or 270 µg/kg/dose) or vehicle were administered intragastrically once a day for 7 days before induction of AP. The effect of warfarin on the severity of AP was assessed 6 h after pancreatic reperfusion. The assessment included histological, functional, and biochemical analyses. Pretreatment with warfarin given at a dose of 90 or 180 µg/kg/dose increased the international normalized ratio and reduced morphological signs of pancreatic damage such as pancreatic edema, vacuolization of acinar cells, necrosis and the number of hemorrhages. These effects were accompanied by an improvement of pancreatic blood flow and a decrease in serum level amylase, lipase, pro-inflammatory interleukin-1β and plasma level of D-dimer. In contrast, pretreatment with warfarin given at a dose of 270 µg/kg/dose led to an increase in severity of pancreatic damage and biochemical indicators of AP. In addition, this dose of warfarin resulted in deaths in some animals. Pretreatment with low doses of warfarin inhibits the development of AP induced by pancreatic ischemia followed by reperfusion.

## 1. Introduction

Coagulation and inflammation show a close relationship based on positive feedback. Inflammation leads to activation of coagulation, which stimulates the development of inflammation [1,2]. Pro-inflammatory cytokines play an important role in the activation of coagulation cascade leading to the formation of fibrin. This effect is associated, among others, with the overexpression of tissue factor on monocytes and endothelial cells [3,4]. Apart from the promotion of clot formation, inflammation also leads to the reduction in the activity of anticoagulant mechanisms and inhibits fibrinolysis [3,5]. Infection and inflammation lead to activation of coagulation and in severe cases may lead to disseminated intravascular coagulation (DIC) [4].

In the same way, coagulation results not only in fibrin and clot formation, but also stimulates the development of inflammation. Pro-inflammatory effect of coagulation is mainly related to the presence of thrombin, active factor X and the complex of tissue factor (TF) with the active factor VII. Thrombin and the complex of tissue factor with active factor VII bind to protease activated receptors (PARs). PARs are expressed by platelets, endothelial cells and various immune cells, including monocytes, lymphocytes, macrophages, dendritic cells and mast cells [6,7]. Stimulation of PARs leads to the production of free radicals and expression of adhesion proteins in endothelial cells, macrophages and monocytes [1,2,8]. In platelets, stimulation of PARs leads to the release of soluble ligand for CD40 receptor (sCD40L) [9]. Soluble CD40L stimulates tissue factor production and the release of pro-inflammatory cytokines [10]. Moreover, sCD40L, acting on vascular endothelial cells stimulates expression of adhesion proteins, leading to rolling and sticking of leukocytes to the vascular endothelium, and subsequent inflammatory infiltration of tissues [11]. Pro-inflammatory effect of thrombin is also associated with the stimulation of the release of pro-inflammatory interleukin-6 and monocyte chemotactic protein-1 (MCP-1) by epithelial and mononuclear cells [5].

The development of AP is associated with activation of coagulation, and the degree of this activation is associated with AP severity. In mild AP, local scattered thrombosis is observed in pancreatic microcirculation. In severe AP, systemic activation of coagulation may reach the level of DIC [12,13,14]. Laboratory markers of hemostasis are useful for the prediction of AP severity and mortality in this disease [13,14,15].

These observations led to the concept that anticoagulants may be useful in the prevention and treatment of AP. Heparin, a naturally occurring glycosaminoglycan, exhibits an anticoagulant and anti-inflammatory effect [16,17,18]. Heparin prevents coagulation by forming the complex with a plasma α_2_-globulin, antithrombin (AT), previously known as antithrombin III. Antithrombin as a serine protease inhibitor inhibits active factors involved in coagulation, including thrombin, factors IXa, Xa, XIa and XIIa, and the complex of TF with factor VIIa [16,17]. The ability of AT to inhibit serine proteases is markedly enhanced in the presence of heparin or heparin sulfate [17]. Apart from inhibitory effect of heparin on the coagulation cascade, heparin alone or in the complex with either AT or heparin cofactor II also inhibits the activity of trypsin [19,20] and chymotrypsin [20], as well as prevents the transformation of inactive trypsinogen to active trypsin [21,22].

Experimental animal studies have shown that pretreatment or treatment with heparin inhibit the development of AP and accelerates the recovery from this disease [23,24,25,26,27]. Also, some clinical studies have reported that heparin may prevent the development of AP and exhibit its therapeutic effect in the course of the disease [28,29,30,31]. Moreover, heparin given together with insulin is recommended in the treatment of patients with AP evoked by hyperlipidemia [32,33,34]. Low molecular weight heparin has been also shown to be effective in the prevention of encephalopathy in the course of severe AP [35].

In harmony with those findings are the observations that the inhibition of coagulation by administration of low doses of acenocoumarol inhibits the development of AP induced by ischemia/reperfusion [36] or cerulein [37] as well as exhibits a therapeutic effect in the course of the disease [38].

Acenocoumarol belongs to 4-hydroxycoumarins, a class of vitamin K antagonist anticoagulant drugs. The primary mechanism of anticoagulant activity of 4-hydroxycoumarin drugs is the inhibition of vitamin K epoxide reductase, an enzyme that restores the active form of vitamin K in the body by recycling the inactive alkoxide-epoxide form of vitamin K back to its active hydroquinone form [39]. Acenocoumarol, warfarin and phenprocoumon are the most commonly prescribed 4-hydroxycoumarins in Europe [40]. Warfarin currently holds a dominant position in this group of drugs due to its long half-life and stable anticoagulant effect. All 4-hydroxycoumarins have a single, chiral center with an *S*- or an *R*-enantiomeric form. The drugs are prepared as racemic mixtures consisting of 50% of each enantiomer. Half-life of warfarin varies from 24–33 h for *S*-warfarin to 35–58 h for *R*-warfarin. Acenocoumarol has a shorter half-life. The *S*-enantiomer of acenocoumarol is more active, however, it has a short half-life of 1.8 h. Therefore, the anticoagulant effect of acenocoumarol is mainly related to the *R*-enantiomer with a half-life of 6.6 h [40]. The quality of anticoagulation with a vitamin K antagonist is assessed by the percentage of time when the international normalized ratio (INR) is within the therapeutic range. When the INR is below the therapeutic range, the risk of thromboembolic events remains high, whereas an increase of INR above the therapeutic range is associated with the risk of bleeding [40]. Previous studies have shown that treatment with acenocoumarol is associated with higher risk of unstable response to oral anticoagulation in comparison to treatment with warfarin [41,42]. Moreover, therapy with warfarin requires fewer assays for treatment monitoring [41]. Changing acenocoumarol to warfarin in patients with unstable anticoagulation significantly improves keeping the INR within the therapeutic range [43].

Apart from the anticoagulant effect, coumarins also affect other body functions. Previous studies have showed that coumarin and its 3,4 dihydroquinolinone derivatives present an antidepressant effect [44]; whereas, fraxetin (7,8-dihydroxy-6-methoxy coumarin) exhibits hepatoprotective effect through inhibiting inflammation and apoptosis of hepatocytes, as well as preventing liver fibrosis in carbon tetrachloride-induced liver injury [45]. Moreover, the antifibrotic and anti-inflammatory drug, pirfenidone was found to be able to inhibit the development of l-arginine-induced AP [46]. These observations bring additional support for the concept that warfarin may be useful in the treatment of AP. Therefore, the purpose of the current research was to determine the impact of pretreatment with warfarin, an oral vitamin K antagonist, on the development of ischemia/reperfusion-induced AP in rats. To the best of our knowledge, it is the first research in this field. Because our research was a preliminary study, we chose a model in which AP is caused by primary vascular mechanism.

## 2. Results

### 2.1. The International Normalized Ratio

**Figure 1:**
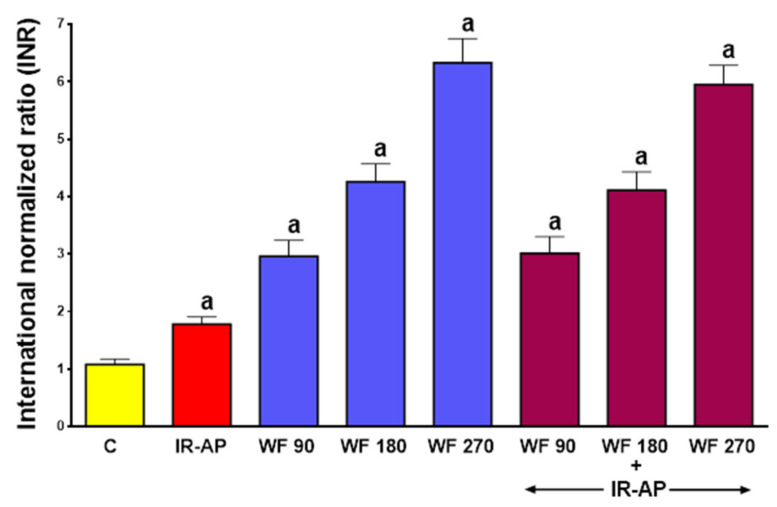
Impact of ischemia/reperfusion-induced acute pancreatitis (IR-AP) or/and pretreatment with warfarin given at a dose of 90, 180 or 270 µg/kg/dose (WF 90, WF 180 or WF 270) on the international normalized ratio (INR). Mean ± SEM. *n* = 10 animals in each experimental group. ^a^ *p* < 0.05 compared to control (C).

In control saline-treated rats without induction of AP, the prothrombin time measured as the international normalized ratio (INR) reached a value of 1.08 ± 0.09 (mean ± SEM) (Figure 1). 

In sham-operated control rats without induction of AP, warfarin administered for 7 days at a dose of 90, 180 or 270 μg/kg/dose, significantly increased the INR to 2.96 ± 0.28 (*p* < 0.05), 4.26 ± 0.31 (*p* < 0.05) and 6.33 ± 0.42 (*p* < 0.05), respectively. In rats pretreated with saline, induction of AP significantly increased the INR to 1,78 ± 0.13 (*p* < 0.05). In rats treated with warfarin prior to AP induction, the INR reached levels similar to those observed in rats pretreated with warfarin without induction of AP (Figure 1).

### 2.2. Histological Examination

In control saline-pretreated sham operated rats without induction of AP, histological examination showed correct structure of the pancreas without any damage (Table 1, Figure 2.1). In half of the rats pretreated with warfarin at the doses used without induction of AP, morphological images showed no pancreatic edema or other damage. In the second half of the animals in this experimental group, intralobular edema was observed. In addition, single hemorrhagic foci were seen in half of animals treated with warfarin at the highest dose of 270 μg/kg/dose, (Figure 2.5).

Pancreatic ischemia followed by a 6 h reperfusion led to the development of acute hemorrhagic pancreatitis in all rats subjected to this procedure. Histological images of the pancreases showed moderate interlobular and moderate intralobular edema accompanied with moderate perivascular and scarce diffuse leukocytic infiltration. Vacuolization of acinar cells was observed in about half of cases and involved less than 25% of those cells. Necrosis was observed in all cases of AP and involved less than 15% of acinar cells. Hemorrhage was ranged from limited to 1–5 foci per slide (Table 1, Figure 2.2). Pretreatment with warfarin given at a dose of 90 or 180 μg/kg/dose prior to AP induction inhibited the development of morphological signs of pancreatic damage observed in histological examination (Table 1, Figure 2.6,7).

Those doses of warfarin reduced pancreatic edema, vacuolization of acinar cells and number of hemorrhages foci. Moreover, warfarin given at a dose of 180 μg/kg/dose reduced the number of cells affected by necrosis. In contrast, administration of warfarin at a dose of 270 μg/kg/dose did not show any protective effect on the pancreas, but increased pancreatic edema, vacuolization of acinar cells and number of hemorrhagic foci (Table 1, Figure 2.8).

Those doses of warfarin reduced pancreatic edema, vacuolization of acinar cells and number of hemorrhages foci. Moreover, warfarin given at a dose of 180 μg/kg/dose reduced the number of cells affected by necrosis. In contrast, administration of warfarin at a dose of 270 μg/kg/dose did not show any protective effect on the pancreas, but increased pancreatic edema, vacuolization of acinar cells and number of hemorrhagic foci (Table 1, Figure 2.8).

### 2.3. Serum Activity of Pancreatic Digestive Enzymes

**Figure 3:**
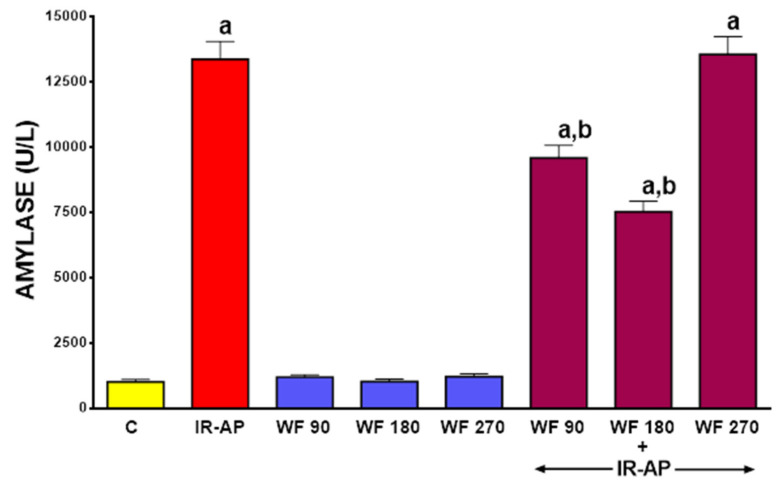
Impact of ischemia/reperfusion-induced acute pancreatitis (IR-AP) or/and pretreatment with warfarin given at a dose of 90, 180 or 270 µg/kg/dose (WF 90, WF 180 or WF 270) on serum amylase activity. Mean ± SEM. *n* = 10 animals in each experimental group. ^a^ *p* < 0.05 compared to control (C), ^b^ *p* < 0.05 compared to IR-AP.

Serum activity of amylase in control saline-treated sham-operated rats was about 1,000 U/L (Figure 3). In rats pretreated with warfarin without induction of AP, serum activity of amylase tended to increase, but this effect was statistically insignificant. Induction of AP led to an approximately 13-fold increase in serum activity of amylase. Pretreatment with warfarin given at a dose of 90 or 180 µg/kg/dose partly but significantly reversed the AP-induced increase in serum activity of amylase (*p* < 0.05 in both cases). This effect was more pronounced after warfarin was given at a dose of 180 µg/kg/dose, but the difference between effect of warfarin at a dose of 90 and 180 µg/kg/dose was not significant (*p* > 0.05). Pretreatment with warfarin given at a dose of 270 µg/kg/dose did not exhibit any inhibitory effect on serum activity of amylase in rats with AP(*p* > 0.05) (Figure 3).

**Figure 4:**
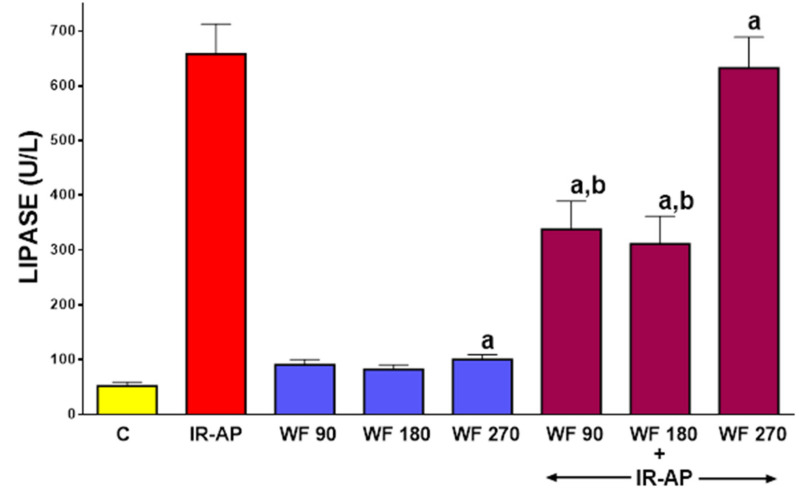
Impact of ischemia/reperfusion-induced acute pancreatitis (IR-AP) or/and pretreatment with warfarin given at a dose of 90, 180 or 270 µg/kg/dose (WF 90, WF 180 or WF 270) on serum lipase activity. Mean ± SEM. *n* = 10 animals in each experimental group. ^a^ *p* < 0.05 compared to control (C), ^b^ *p* < 0.05 compared to IR-AP.

In control saline-treated sham-operated rats without induction of AP, serum activity of lipase was 52.2 ± 6.7 U/L (mean ± SEM) (Figure 4). In rats without induction of AP, pretreatment with warfarin at the doses given led to a slight increase in serum activity of lipase and in the case of warfarin given at a dose of 270 µg/kg/dose this effect was statistically significant (*p* < 0.05). AP induction increased serum activity of lipase to 658.0 ± 54.4 U/L (*p* < 0.05). Pretreatment with warfarin given at a dose of 90 or 180 µg/kg/dose significantly reversed this increase (*p* < 0.05 in both cases). In contrast, pretreatment with warfarin given at a dose of 270 µg/kg/dose was without effect on the serum activity of lipase in rats with AP (*p* > 0.05) (Figure 4).

### 2.4. Serum Level of Interleukin-1β (IL-β)

**Figure 5:**
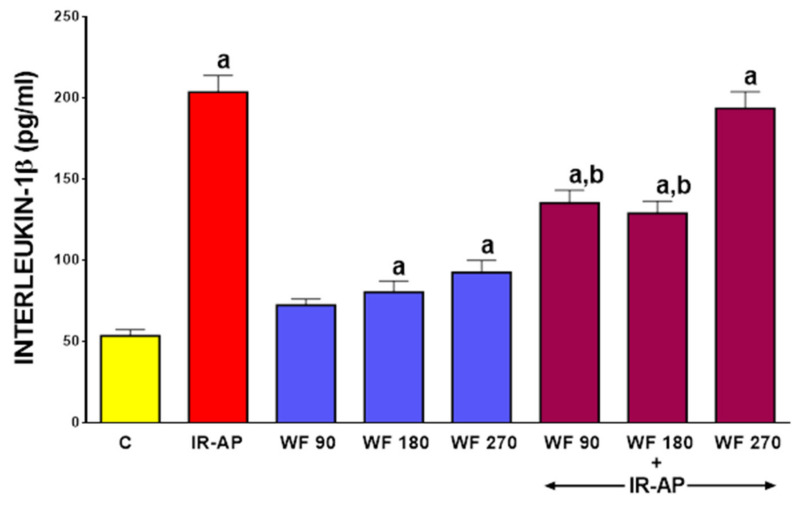
Impact of ischemia/reperfusion-induced acute pancreatitis (IR-AP) or/and pretreatment with warfarin given at a dose of 90, 180 or 270 µg/kg/dose (WF 90, WF 180 or WF 270) on serum concentration of interleukin-1β. Mean ± SEM. *n* = 10 animals in each experimental group. ^a^ *p* < 0.05 compared to control (C), ^b^ *p* < 0.05 compared to IR-AP.

Concentration of pro-inflammatory IL-1β in serum of control saline-treated sham-operated rats was 53.4 ± 3.9 pg/mL (mean ± SEM) (Figure 5). 

In those rats, pretreatment with warfarin slightly increased serum level of IL-1β and this effect was statistically significant in rats pretreated with warfarin given at a dose of 180 and 270 µg/kg/dose (*p* < 0.05 in both cases). Induction of AP led to an approximately 4-fold increase in the serum level of IL-1β of rats pretreated with saline. Pretreatment with warfarin given at a dose of 90 or 180 µg/kg/dose significantly reduced the increase in the serum level of IL-1β in rats with AP (*p* < 0.05 in both cases). Pretreatment with warfarin administered at a dose of 270 µg/kg/dose was without any marked effect on serum level of IL-1β in rats with AP (*p* > 0.05) (Figure 5).

### 2.5. Pancreatic Blood Flow

**Figure 6:**
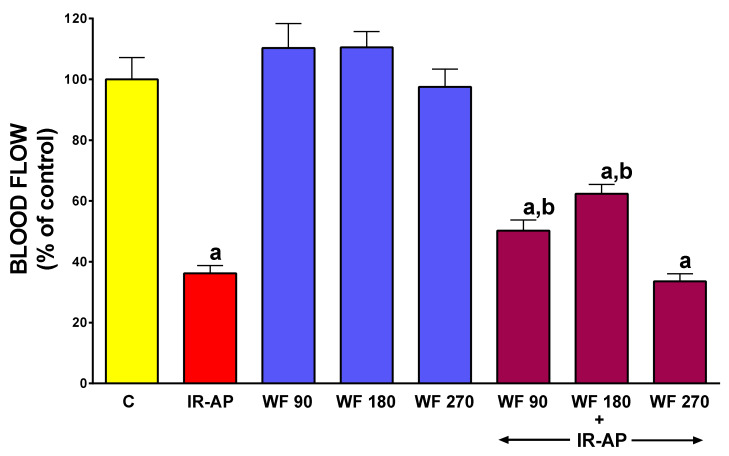
Impact of ischemia/reperfusion-induced acute pancreatitis (IR-AP) or/and pretreatment with warfarin given at a dose of 90, 180 or 270 µg/kg/dose (WF 90, WF 180 or WF 270) on pancreatic blood flow. Mean ±SEM. *n* = 10 animals in each experimental group. ^a^ *p* < 0.05 compared to control (C), ^b^ *p* < 0.05 compared to IR-AP.

In rats without induction of AP, administration of warfarin at given doses was without significant effect on pancreatic blood flow (Figure 6). Induction of AP reduced pancreatic blood flow by about 64%. Pretreatment with warfarin given at the dose of 90 or 180 µg/kg/dose partly, but significantly reversed the pancreatitis-evoked reduction in pancreatic blood flow (*p* < 0.05 and *p* < 0.005, respectively). Pretreatment with a higher dose of warfarin, 270 µg/kg/dose did not improve pancreatic blood flow in rats with AP (Figure 6).

### 2.6. Plasma Concentration of D-dimer

**Figure 7:**
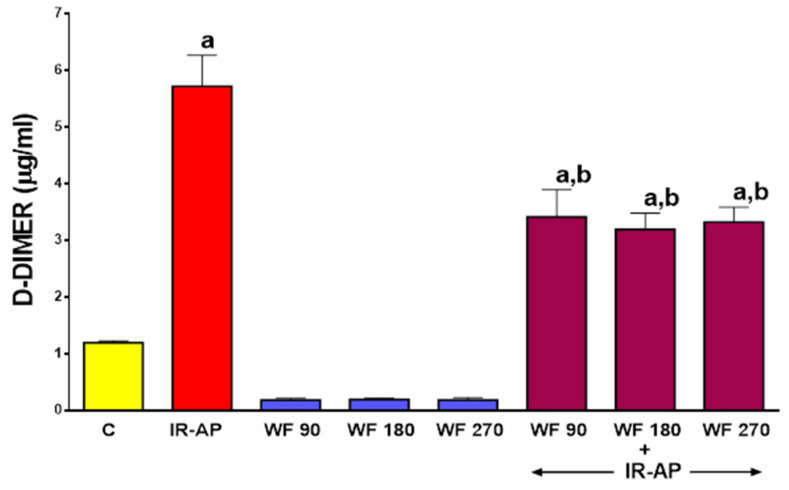
Impact of ischemia/reperfusion-induced acute pancreatitis (IR-AP) or/and pretreatment with warfarin given at a dose of 90, 180 or 270 µg/kg/dose (WF 90, WF 180 or WF 270) on plasma D-Dimer concentration. Mean ± SEM. *n* = 10 animals in each experimental group. ^a^ *p* < 0.05 compared to control (C), ^b^ *p* < 0.05 compared to IR-AP.

In control saline-treated sham-operated rats without induction of AP, plasma level of D-dimer, a fibrin degradation product was 1.19 ± 0.03 µg/mL (mean ± SEM) (Figure 7). Pretreatment with warfarin at given doses insignificantly reduced plasma level of D-Dimer in rats without induction of AP. Induction of AP led to an approximately 5-fold increase in plasma level of D-Dimer. Pretreatment with any given dose of warfarin partly reversed this effect and reduced the serum level of D-Dimer in rats with AP by about 40% (*p* < 0.05 in all cases). (Figure 7).

### 2.7. Animal Mortality During Experiment

Three rats died on the third day of pretreatment with warfarin given at a dose of 270 µg/kg/dose without induction of AP. Autopsy of those rats showed hemorrhage in the abdominal cavity. In the remaining groups, no premature animal death was observed prior to study termination.

## 3. Discussion

As presented in the Introduction, there is a close relationship between coagulation and inflammation. Both of these processes lead to mutual activation [1,2,3,4,5,6,7,8,9,10,11]. This relationship is also observed in case of acute pancreatitis (AP) [12,13,14,15]. Previous studies with heparin [23,24,25,26,27,28,29,30,31,32,33,34,35], acenocoumarol [36,37,38] and antithrombin [47,48,49,50] have shown that inhibition of coagulation exhibits a protective effect on the pancreas and accelerates recovery in the course of AP regardless of the primary cause of the disease. The aim of our present study was to investigate the impact of pretreatment with warfarin on the development of ischemia/reperfusion-induced AP.

Warfarin is a vitamin K antagonist and its anticoagulant effect is induced by inhibiting the activity of vitamin K epoxide reductase, an enzyme that leads to the cyclic conversion of the inactive oxidized vitamin K epoxide to its active reduced hydroquinone form [39,51]. Reduced form of vitamin K is necessary for the γ-carboxylation of glutamate residues of vitamin K-dependent coagulation factor II (prothrombin), VII, IX and X, as well as anticoagulant factors such as protein S, C and Z [16,51]. Vitamin K antagonists create deficiency of the reduced form of the vitamin leading to the inhibition of carboxylation and maturation of clotting factors. Partly carboxylated or non-carboxylated clotting factors are biologically inactive and are known as proteins induced by vitamin K absence (PIVKA) [52]. Carboxylation is necessary for calcium-dependent changes in coagulation factor conformation that promotes binding of cofactors on the phospholipid surface. Vitamin K antagonists inhibit synthesis of mature form of prothrombin and coagulation factor VII, IX and X, and therefore inhibit coagulation cascade in the internal and external pathway. Moreover, treatment with vitamin K antagonists, warfarin and acenocoumarol was shown to increase clot permeability with enhanced efficiency of fibrinolysis [53]. Anticoagulated patients with atrial fibrillation with increased clot permeability have an increased risk of minor bleeding, whereas patients with lower clot permeability have increased risk of ischemic stroke or transient ischemic attack and major bleeding [53,54].

Our study showed that pretreatment with low doses of warfarin inhibits the development of acute pancreatitis (AP) evoked by pancreatic ischemia followed by reperfusion. Warfarin given at a dose of 90 and especially 180 µg/kg/dose reduced the manifestation of histological and biochemical signs of pancreatic damage.

Our present study showed that induction of AP significantly increased the INR. This finding indicates that development of AP activates coagulation leading to consumptive coagulopathy and this observation is in agreement with previous reports [12,13,14]. Moreover, the concept of consumptive coagulopathy is additionally supported by our current observation that induction of AP led to an approximately 5-fold increase in plasma D-Dimer concentration. D-Dimer is the product of stabilized fibrin degradation and for this reason it is related to the activation of coagulation and amount of fibrin in the body [13]. Warfarin treatment leads to reduction in the synthesis of mature coagulation factors and this is the reason for the increase in INR, which we observed after administration of warfarin in animals without AP. This effect was associated with reduction in D-Dimer concentration. This observation additionally indicates that warfarin reduces formation of fibrin in the circulation. In animals pretreated with warfarin prior to AP induction, INR values reached levels similar to those observed in animals pretreated with warfarin without AP induction. Moreover, pretreatment with warfarin prior to AP induction reduced the level of plasma D-Dimer concentration. These both observations indicate that pretreatment with warfarin inhibits the pancreatitis-evoked coagulation and intravascular formation of fibrin.

In our present study, the induction of AP led to an approximately 13-fold increase in serum activity of amylase and lipase. In physiological conditions, amylase and lipase are released at the apical part of the pancreatic acinar cells and from the lumen of pancreatic acini through pancreatic ducts reach the duodenum. In AP, amylase and lipase are ectopically released by basolateral parts of acinar cells into the interstitial space of the pancreas and thence into the blood. In clinical practice, the increase in serum activity of pancreatic amylase and lipase are markers of AP development with high sensitivity and specificity [55]. Moreover, experimental [56,57,58,59] and some clinical [60] studies suggest that plasma level of amylase and lipase reflect the severity of AP. Active pancreatic enzymes, especially proteases, present in pancreatic interstitial space and circulation lead to local and remote organ injury. Moreover, they up-regulate the adhesion molecules expression on the cell membrane of leukocytes and endothelial cells, leading to the disturbance of organ microcirculation inflammatory infiltration [61]. Our present study showed that pretreatment with warfarin given at a dose of 90 or 180 µg/kg/dose significantly reduced the pancreatitis-evoked increase in serum activity of amylase and lipase. This observation indicates that pretreatment with warfarin may inhibit the development of AP induced by pancreatic ischemia followed by reperfusion.

Our current study also showed that pretreatment with warfarin given at a dose of 90 or 180 µg/kg/dose reduced the development of morphological symptoms of AP. Warfarin prevented the development of vacuolization of acinar cells, decreased the number of hemorrhagic foci, as well as reduced the degree of pancreatic edema and inflammatory infiltration. Moreover, warfarin given at a dose of 180 µg/kg/dose decreased necrosis of pancreatic cells.

Inflammatory leukocyte infiltration plays an important role in the development of inflammation and progression of its severity. The development of AP is associated with induction of the inflammatory response with accumulation of leukocytes within the pancreas. Activated leukocytes adhere to vascular endothelial cells, infiltrate pancreatic tissue, produce pro-inflammatory cytokines in the pancreas and increase local and systemic levels of these cytokines. Early and persistent activation of inflammatory cells and release of pro-inflammatory cytokines is responsible for the severity of local and systemic inflammatory response in AP, as well as the development of systemic inflammatory response syndrome (SIRS) and multiple organ dysfunction syndrome (MODS), leading to the development of severe AP [62,63,64]. In acute pancreatitis, pro-inflammatory cytokines such as Interleukin-1β (IL-1β), IL-6 and tumor necrosis factor-α (TNF-α) are primarily produced within the pancreas and subsequently within distant organs. IL-1β plays an essential role in the release of other members of pro-inflammatory cytokine cascade and the induction of systemic acute phase response [65]. The increase in pro-inflammatory interleukins level is a result of the development of inflammation, but simultaneously these cytokines also play a key role in the development of inflammation as its mechanism. In line with this concept are the results of experimental studies, showing that effective anti-inflammatory treatment in AP is associated with reduction in the level of IL-1β in the pancreas and serum [66,67,68,69]. Moreover, experimental studies have showed that administration of IL-1 receptor antagonist decreases severity of experimental acute pancreatitis [70], whereas overexpression of IL-1β in transgenic mice results in chronic pancreatitis [71]. Also some clinical studies even suggest that the genetic polymorphism of interleukin 1 receptor antagonist may be associated with increased risk of AP development [72]. The relationship between the level of pro-inflammatory cytokines and the severity of the disease was also found in other organs. For example in the brain, it has been well known that acute infections, as well as chronic exposure to infectious agents may elevate the risk of stroke. Observations performed in patients with ischemic stroke indicate that the expression of pro-inflammatory agents and markers on the first day of the stroke has an important prognostic value for the severity of brain damage and post-stroke deficit [73].

Our current study is in agreement with the above presented data. Induction of AP led to inflammatory infiltration of pancreatic tissue associated with an increase in serum concentration of pro-inflammatory IL-1β. Pretreatment with warfarin given at a dose of 90 or 180 µg/kg/dose reduced pancreatic inflammatory infiltration and significantly reversed the increase in serum IL-1β level in rats with induction of AP. These data additionally indicate that pretreatment with warfarin inhibits the development of AP evoked with pancreatic ischemia followed by reperfusion.

Adequate blood flow for organ metabolism plays a key role in maintaining organ integrity. Numerous clinical and experimental studies have showed that pancreatic ischemia may be the primary cause of AP, as well as plays an essential role in the development of severe forms of the disease [74,75,76]. The early disturbance of pancreatic circulation is also observed in AP caused by other, primarily non-vascular mechanisms [77,78]. Moreover, previous studies indicated that the improvement of pancreatic blood flow inhibits the development of AP and accelerates the recovery [79,80]. Similar protective and recovery promoting effect of blood flow improvement has also been shown in other organs of the digestive system such as the oral cavity [81], stomach [82,83], duodenum [84,85] and colon [86,87]. Our present study confirms previous findings that induction of AP leads to the reduction in pancreatitis blood flow. A new finding of our study is the observation that pretreatment with warfarin given at a dose of 90 or 180 µg/kg/dose significantly improves pancreatic blood flow in animals with AP development. This observation indicates that protective effect of warfarin in ischemia/reperfusion-induced AP acute pancreatitis involves improvement of pancreatic microcirculation and it is the evidence that pretreatment with this coumarin exhibits the protective and anti-ischemic effect on the pancreas.

Studies concerning early events of acute pancreatitis and new concepts of treatment cannot be performed on humans due to the ethical reason, Animal models of AP have been created to solve this problem. Mice and rats are commonly used in animal models of acute pancreatitis because studies on these animals are better standardized and cheaper. On the other hand, however, it should be remembered that although mice and rats are most commonly used in animal models of AP, the results obtained in these models do not necessarily fully correspond to clinical condition [88]. Rodent pancreas has a different anatomy than human pancreas. There are also differences in functional and biochemical parameters between rodents and humans. In the most countries, the target INR range for patients with fibrillation or venous thromboembolismis is from 2.0 to 3.0. For some indications such as prosthetic heart valves a higher target range is recommended, from 2.5 to 4.0 [40]. In our present study, the best protective effects were observed after warfarin given at a dose of 180µg/kg/dose. This effect was associated with an increase in INR above 4. Lower doses of warfarin led to a less prolongation of INR and their efficiency in preventing pancreatitis was lower. The needs to obtain the INR value above 4 may be a result of species differences between humans and rats, as well as seems to be a result of characteristic of the pancreatitis model used in our study. AP was induced by pancreatic ischemia lasting for 30 min followed by reperfusion. This led to the development of ischemia/reperfusion-induced damage of the pancreas, as well as activation of clotting. It should be noted, however, that the presented results were obtained in preliminary studies on the effect of warfarin on the development of AP and the purpose of these studies was to determine whether warfarin can protect the pancreas. Therefore at this stage of the study, the determination of warfarin doses and INR values in possible clinical trials seems premature. Moreover, for clinical point of view, the protective effect of warfarin preventing the development of AP has a minimal value in the treatment of this disease in humans. Eventually, it could be useful before procedures that may lead to the development of AP, for example before endoscopic retrograde cholongiopancreatography. On the other hand, there are retrospective studies showing that preexisting systemic anticoagulation prevents the development of AP in humans and reduces the severity of this disease. During Digestive Disease Week 2019 in San Diego, Dr. Yan Bi presented results from 442,535 patients with AP. Of these, 12,735 were on systemic anticoagulation prior to the onset of AP [89]. The study were performed using data from the National Inpatients Sample 2014. Dr. Yan Bi and co-workers found that patients on systemic anticoagulation exhibit decreased odds of AP occurrence, mortality, shock, acute kidney injury, multiorgan failure and hospital charges compared to patients who are not on systemic anticoagulation [90]. These data are in harmony with our current results and provide the additional evidence conforming the role of coagulation in AP.

It should be noted, however, that the eventual therapeutic effect of warfarin in the course of AP would have is more important. Experimental studies addressing this topic should be the subject of future studies.

Our current research has shown that pre-treatment with warfarin inhibits ischemia/reperfusion-induced AP development. It should be noted, however, that this therapy was associated with some risks and side effects. The best protective effect was observed after administration of warfarin at a dose of 180 µg/kg/dose. A lower dose was less effective. However, warfarin given at a dose of 180 µg/kg/dose increased INR to 4.26. This value of INR is associated with an increased risk of bleeding [40]. Moreover, all the used warfarin doses led to a slight pancreatic edema in rats without AP. The highest dose of warfarin led to the development of hemorrhagic foci in the pancreas of animals without AP induction. In addition, administration of warfarin at a dose of 180 or 270 µg/kg/dose caused a small, but statistically significant increase in serum level of IL-1β in animals without AP. Moreover, warfarin given at the dose of 270 µg/kg/dose led to a significant increase in lipase activity in rats without AP. The most important complication in rats treated with the highest dose of warfarin was the death of three animals due to intra-abdominal hemorrhage in a group without AP induction. In case of animals with induction of AP, warfarin given at the dose of 90 or 180 µg/kg/dose exhibited a significant protective effect. These observations indicate that warfarin exhibits some preventive effect in ischemia reperfusion-induced AP, but this therapy is associated with increased risk of bleeding. In addition, warfarin used to prevent the development of AP has a small therapeutic window. Warfarin given at a dose of 180 µg/kg/dose exhibited a maximal protective effect, while warfarin given at a dose of 270 µg/kg/dose had no therapeutic effect. When attempting to use warfarin to prevent the development of AP in clinical conditions, some improvement in therapeutic effects could probably be achieved by conducting a genetic consultation and determining an individual dose for each patient. The carriers of common genetic variants in cytochrome P-450 2C9 (CYP2C9) and vitamin K epoxide reductase complex subunit 1 (VKORC1) required about 20% lower dosage than the wild-type [40,51,91].

## 4. Materials and Methods

### 4.1. Animals and Treatment

All studies followed an experimental protocol approved by the Committee for Research and Animal Ethics of the Jagiellonian University and the Local Commission of Ethics for the Care and Use of Laboratory Animals in Cracow (Permits Numbers: 25/2016 released on 20 July 2016 and 95/2017 released on 20 December 2017).

Studies were carried out on 83 male Wistar rats weighing 200–220 g, which were housed in cages in a windowless colony room. We originally used 80 rats, but three rats died on the third day of pretreatment with warfarin given at a dose of 270 µg/kg/dose without induction of acute pancreatitis (AP). Autopsy of those rats showed hemorrhage in the abdominal cavity. For this reason, 3 additional rats were used to reach 10 observations in each experimental group. The temperature in the colony room was adjusted at 22 ± 1 °C with relative humidity of 50 ± 10%, and 12–12 h light-dark photoperiod. During the study animals had free access to food and water, apart from the 16 h before the induction of AP, when rats were fasted with free access to water. Following a one-week period of acclimation to their new environment, rats were randomly assigned to 8 experimental groups, as follows: (1) saline-pretreated and sham-operated rats (control group); (2) rats pretreated with saline before the development of ischemia/reperfusion-induced AP; (3) rats pretreated with warfarin given at a dose of 90 µg/kg/dose before a sham-operation; (4) rats pretreated with warfarin given at a dose of 180 µg/kg/dose before a sham-operation; (5) rats pretreated with warfarin given at a dose of 270 µg/kg/day before a sham-operation; (6) rats pretreated with warfarin given at a dose of 90 µg/kg/dose before the development of ischemia/reperfusion-induced AP; (7) rats pretreated with warfarin given at a dose of 180 µg/kg/dose before the development of ischemia/reperfusion-induced AP; (8) rats pretreated with warfarin given at a dose of 270 µg/kg/dose before the development of ischemia/reperfusion-induced AP. The study was terminated 6 h after the initiation of pancreatic reperfusion or sham-operation.

Saline or warfarin (Warfin. Orion Corporation, Espoo, Finland) were administered intragastrically once a day for 7 days before induction of AP or sham-operation. Warfarin was given at a dose of 90, 180 or 270 μg/kg/dose. Doses of warfarin were selected according to the doses used in the clinics. Clinically, initial dose of warfarin in adults is 5–10 mg/day. For a 50 kg person, it gives 100–200 µg/kg/day. In 75 kg person, it gives 66.6–133.3 μg/kg/day. In 100 kg person, it gives 50–100 μg/kg/day.

Before induction of AP or sham-operation rats were anesthetized with ketamine (Bioketan, Vetoquinol Biowet, Gorzów Wielkopolski, Poland; 50 mg/kg intraperitoneally). AP was induced by pancreatic ischemia followed by reperfusion as described previously in details [92]. Briefly, after a longitudinal laparotomy the splenic inferior artery was clamped down by microvascular clips to induce ischemia in the splenic region of the pancreas. Thirty minutes later, microvascular clips were removed to obtain pancreatic reperfusion. The abdominal cavity for the time of reperfusion was closed by suture. In sham-operated control rats, longitudinal laparotomy and mobilization of the celiac artery without clamping any artery was performed. After suture of the abdominal cavity, all animals were injected subcutaneously with tramadol (Poltram 100, Polpharma, Starogard Gdański, Poland) given subcutaneously at a dose of 1 mg/kg/dose to minimize pain and distress, and received 5 mL of Ringer’s solution for the supply of fluids lost during the surgery and postoperative period.

### 4.2. Determination of Pancreatic Blood Flow

At the end of the study, after 6-h pancreatic reperfusion or 6 h after sham-operation, the animals were again anesthetized with ketamine. After opening the abdominal cavity and exposure the pancreas, blood flow in splenic region of the pancreas was measured using a laser Doppler flowmeter (PeriFlux 4001 Master Monitor, Perimed AB, Järfälla, Sweden), as described previously in detail [93,94]. Data were presented as percentage of pancreatic blood flow obtained in sham-operated saline-treated rats without induction of AP.

### 4.3. Biochemical Analysis

After the measurement of pancreatic blood flow, blood samples were taken from the abdominal aorta. The prothrombin time measured as international normalized ratio (INR) was determined in fresh blood, using Alere INRatio^®^ 2 PT/INR Monitoring Systems and Alere INRatio^®^ PT/INR Monitoring System Test Strips (Alere San Diego, Inc, San Diego, CA, USA).

Plasma D-Dimer concentration was determined using an immunoturbidimetric assay (Innovance D-Dimer Assay, Siemens Healthcare GmbH, Marburg, Germany) on automatic coagulation analyzer BCS XP System (Siemens Healthcare Diagnostics, Erlangen, Germany).

Serum lipase and amylase activity was determined with a Kodak Ectachem DT II System analyzer (Eastman Kodak Company, Rochester, NY, USA) using Lipa and Amyl DT Slides (Vitros DT Chemistry System, Johnson & Johnson Clinical Diagnostic, Inc., Rochester, NY, USA).

Serum concentration of interleukin-1β (IL-1β) was measured using the Rat IL-1 beta ELISA Kit (Biorbyt Ltd., Cambridge, UK).

### 4.4. Histological Examination of Pancreatic Damage

Samples of pancreatic tissue were fixed in 10% buffered formalin. Slides were stained with hematoxylin and eosin, and were examined by two pathologists who were unaware of the treatment given. As described previously [95], the following histological signs of pancreatic damage were examined (grading from 0 to 3):

(1) pancreatic edema (0—no edema, 1—interlobar edema, 2—interlobar and moderate intralobular edema, 3—severe interlobular and intralobular edema);
(2) leukocyte inflammatory infiltration (0—absent, 1—scarce perivascular infiltration, 2—moderate perivascular and scarce diffuse infiltration, 3—abundant diffuse infiltration);
(3) vacuolization of acinar cells (0—absent, 1—involving less than 25% of acinar cells, 2—involving from 25% to 50% acinar cells, 3—involving more than 50% of acinar cells);
(4) necrosis of acinar cells (0—absent, 1—involving less than 15% of acinar cells, 2—involving from 15% to 35% acinar cells, 3—involving more than 35% of acinar cells);
(5) hemorrhage (0—absent, 1—from 1 to 2 foci per slide, 2—from 3 to 5 foci per slide, 3—more than 5 foci per slide).

Results of histological examination were expressed as a predominant histological score (mode) of each sign of pancreatic damage in each experimental group.

### 4.5. Statistical Analysis

Statistical analysis was made by analysis of variance followed by Tukey’s multiple comparison test using GraphPadPrism (GraphPad Software Inc., San Diego, CA, USA). The results were presented as means ± SEM. Each experimental group consisted of 10 animals. The difference with a *P* value less than 0.05 was considered significant.

## 5. Conclusions

In conclusion, we can say that pretreatment with warfarin inhibits the development of acute pancreatitis evoked by pancreatic ischemia followed by reperfusion. These findings indicate that activation of coagulation is involved in the mechanism of acute pancreatitis development in this model of disease and warfarin may bring some benefits in the treatment of acute pancreatitis in humans. However, it should be noted that treatment with warfarin in acute pancreatitis may be associated with some risks and side effects. The biggest problems are the risk of bleeding and the narrow therapeutic window. Some improvement in therapeutic effects of warfarin can probably be achieved with conducting a genetic consultation and determining the individual dose for each patient. However, further research in this field is needed.

## Figures and Tables

**Figure 2:**
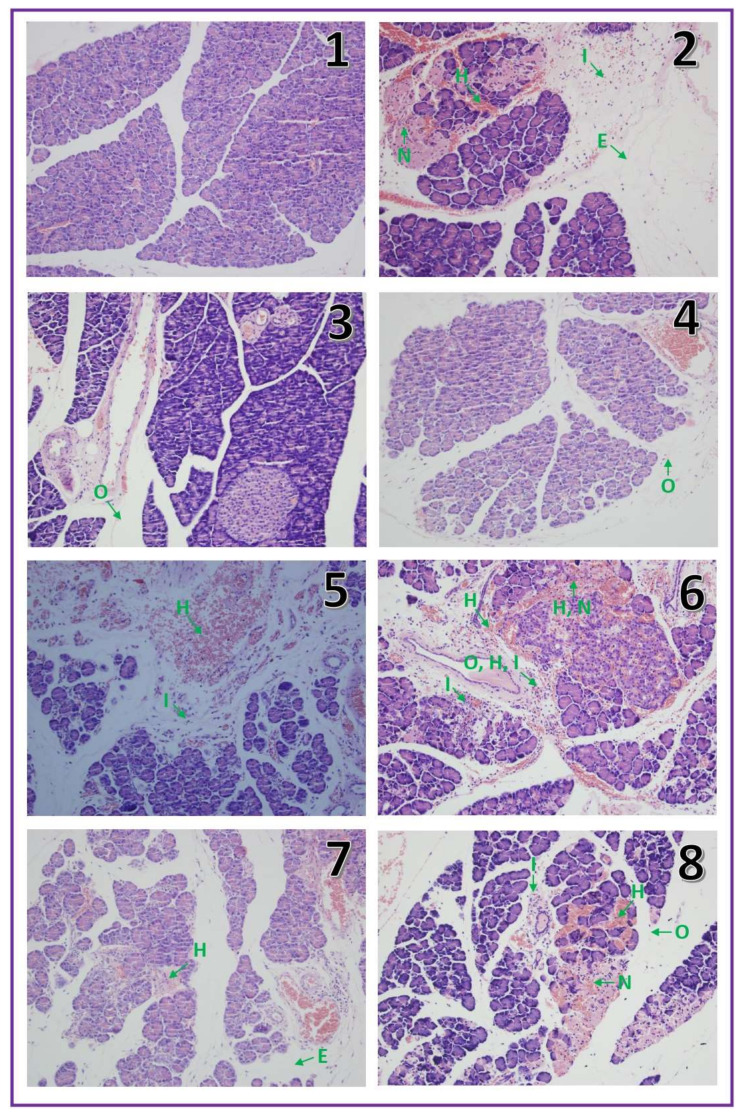
Representative morphological features of the pancreas observed in sham-operated saline-treated control rats (**1**), rats with ischemia/reperfusion-induced acute pancreatitis (**2**), rats treated with warfarin at a dose of 90 µg/kg/dose without induction of acute pancreatitis (**3**), rats treated with warfarin at a dose of 180 µg/kg/dose without induction of acute pancreatitis (**4**), rats treated with warfarin at a dose of 270 µg/kg/dose without induction of acute pancreatitis (**5**), rats pretreated with warfarin at a dose of 90 µg/kg/dose before induction of acute pancreatitis (**6**), rats pretreated with warfarin at a dose of 180 µg/kg/dose before induction of acute pancreatitis (**7**), rats pretreated with warfarin at a dose of 270 µg/kg/dose before induction of acute pancreatitis (**8**); The arrow with E means edema, with I: inflammatory infiltration, with V: vacuolization, with N: necrosis with H: hemorrhages. Hematoxylin-eosin stain, original magnification 200×.

**Table 1:** Influence of ischemia/reperfusion-induced acute pancreatitis (IR-AP) and pretreatment with warfarin given at the dose of 90, 180 or 270 μg/kg/day, applied alone or in their combination (warfarin plus IR-AP) on morphological signs of pancreatic damage.

	Edema (0–3)	Inflammatory infiltration (0–3)	Vacuolization (0–3)	Necrosis (0–3)	Hemorrhages (0–3)
Control	0	0	0	0	0
IR-AP	2	2	0–1	1	1–2
Warfarin 90	0–1	0	0	0	0
Warfarin 180	0–1	0	0	0	0
Warfarin 270	0–1	0	0	0	0–1
Warfarin 90 + IR-AP	1–2	1	0	1	1
Warfarin 180 + IR-AP	1–2	1	0	0–1	1
Warfarin 270 + IR-AP	2–3	2	1	1	2

Numbers represent the predominant histological grading in each group.
